# Why snakebite patients in Myanmar seek traditional healers despite availability of biomedical care at hospitals? Community perspectives on reasons

**DOI:** 10.1371/journal.pntd.0006299

**Published:** 2018-02-28

**Authors:** Eliza Schioldann, Mohammad Afzal Mahmood, Mya Myitzu Kyaw, Dale Halliday, Khin Thida Thwin, Nyein Nyein Chit, Robert Cumming, David Bacon, Sam Alfred, Julian White, David Warrell, Chen Au Peh

**Affiliations:** 1 School of Public Health, University of Adelaide, Adelaide, Australia; 2 Project Field Team, Myanmar Snakebite Project, Mandalay, Myanmar; 3 Ministry of Health and Sport, University of Medicine 1 & Yangon Specialist Hospital, Yangon, Myanmar; 4 Regional Department of Public Health, Ministry of Health, Mandalay, Myanmar; 5 School of Public Health, University of Sydney, Sydney, Australia; 6 Emergency Department, Royal Adelaide Hospital, Adelaide, Australia; 7 Toxinology Department, Women’s & Children Hospital, Adelaide, Australia; 8 Nuffield Department of Clinical Medicine, University of Oxford, Oxford, United Kingdom; 9 Department of Renal Medicine, Royal Adelaide Hospital, Adelaide, Australia; Institut de Recherche pour le Développement, BENIN

## Abstract

**Background:**

Snakebite is a major public health problem in many developing countries. Farmers are particularly exposed to snakes, and due to their rural location often experience delays in accessing formal healthcare. The reasons to use traditional healers may include difficulties in accessing formal healthcare, certain beliefs about snakes and snake venom, tradition, and trust in the capacity of traditional healers. Traditional healing, however, may have serious consequences in terms of delays or added complications. There is little in-depth current information about the reasons for its continued use for snakebite. As part of a health services development project to improve health outcomes for snakebite patients, community attitudes to the use of traditional healers were explored in the Mandalay region of Myanmar.

**Methodology & findings:**

With the objective of learning from local communities, information was generated in three communities using participatory appraisal methods with the communities, and focus group discussions with the local healthcare staff. Many snakebite victims in these communities use traditional healing. Reasons include transport difficulties, low cost for traditional healing, inadequacy of anti-snake venom in the formal healthcare sector, and traditional beliefs, as traditional healing practices are rooted in many cultural and traditional factors. The communities reported that even if access to medical care were improved, traditional healing would continue to be used.

**Conclusion:**

These findings point to the need for working with traditional healers for prevention, appropriate first aid and timely access to effective treatment for snakebite.

## Author summary

Snakebite is a major health issue that affects many people, particularly young poor farmers in developing countries in the tropics. Many patients suffer poor outcomes due to inadequate or delayed access to effective treatment. A large number still use traditional healers. Often patients visit traditional healers before accessing formal health care; thus incurring delays in receiving antivenom (AV) needed to treat envenoming. In other cases, traditional healing methods may themselves cause complications. In Myanmar, while most patients now access formal medical care, many also use traditional healers. We consulted communities in three rural areas in the Mandalay region and found that the reasons for using traditional healers include difficulties with transportation, cost, inadequacy of AV in the formal health sector, and trust in traditional healing within the context of longstanding tradition. These findings point to the need for working with the traditional healers as they could be effective agents to encourage prompt use of formal healthcare.

## Introduction

Snakebite is a neglected tropical disease, affecting disempowered rural communities in developing countries. It has been difficult to identify the exact incidence due to inadequate health statistics and the fact that some patients do not seek medical care. In 2008, global annual incidence was estimated as 1.8 million bites [1], whereas in 1998 Chippaux estimated the incidence as high as 5.4 million bites [2]. The global mortality from snakebite likely exceeds 125,000 deaths annually. However, a comprehensive community survey indicated that in India alone, the annual mortality from snakebite exceeded 45,000 [3]. Considering that many patients may not use health services and die before accessing care, the actual number may be much higher [1, 3, 4].

The burden of snakebite is highest in rural areas of the tropics and subtropics of South/ Southeast Asia and sub-Saharan Africa, mainly due to the density and species of venomous snakes present, population density, agriculture base, inadequate public health programs and lack of mechanised farming practices [1, 5, 6].

The only specific treatment for snakebite envenoming is antivenom (AV; “anti-snake-venom”, “ASV”) [7]. However, with less than adequate health literacy, inadequate access to AV and treatment facilities and other reasons, traditional healing continues to be used by a large number of people. Traditional medicine and healing are based on the communities’ past experiences and observations, passed on through generations verbally or in writing [8], and is defined as “the sum total of the knowledge, skill, and practices based on the theories, beliefs, and experiences indigenous to different cultures, whether explicable or not, used in the maintenance of health as well as in the prevention, diagnosis, improvement or treatment of physical and mental illness”[9]. Traditional medicine is used, whether alone or in conjunction with biomedicine (medical care and system based on the principles of Western science), by many people in both developing and developed countries; 80% of people in Africa reported to be users, 40% of all health care in China is reported as traditional care, and 38% to 75% of people in developed countries such as Australia, Canada and France are said to access complementary and alternative medicine [10].

Globally, traditional methods such as tattooing and herbal remedies and other methods including electric shock and suction are still used for snakebite [7, 11, 12]. The reasons listed in the literature for the continuing use of traditional healing include affordability, availability, and cultural familiarity [13, 14]. Unfortunately, a significant number of people continue to die after snakebite. This is often due to severe envenoming, made worse in many cases by delays in obtaining effective medical care. These factors may generate a misperception that formal biomedicine (also known as Western or allopathic medicine) is ineffective [15, 16]. A proportion of snakebites by venomous species are ‘dry bites’, where the bite fails to inject enough venom to cause perceptible clinical effects. Further, many snake species are either non-venomous, or minimally venomous and so unable to cause envenoming. Should the patient seek help from a traditional healer after such a dry bite or non-venomous bite, the patient and the community are likely to mistakenly attribute recovery to the use of traditional medicine.

Snakebite incidence is historically high in Myanmar (15.4/100,000/yr) [17]. 70% of Myanmar’s population resides in rural areas with heavy reliance on subsistence agriculture [18]. Agriculture is a major occupational risk exposing farmers to snakebites. Most venomous bites in Myanmar are attributed to Russell’s Viper, envenoming by which can cause local pain and swelling, coagulopathy, life-threatening hemorrhage, shock, and acute renal failure. Overall, the annual number of snakebite cases, as reported in the national data of snakebite victims who seek care at the government hospitals or health centres fluctuates between 15,000 and 20,000 [19]. A large proportion of these snakebites occur in six high incidence regions i.e. Mandalay, Sagaing, Bago, Magwe, Ayeyarwady and Yangon. According to these health services data, in 2016 there were 16,767 snakebites in Myanmar, out of which 2,566 bites were in Mandalay region. These numbers are probably an underestimation of the magnitude of this important public health issue, as they do not include those victims who use traditional healers only and those who die before seeking care at the government health care centres or hospitals. Myanmar Australia Snakebite project for improved health outcomes for snakebite patients worked closely with the main tertiary hospital in Mandalay region. The hospital admission records and clinical information informed that 965 snakebite victims were admitted in 2016; 68.5% of these 965 suffered from coagulopathy, 63.2% suffered acute kidney injury, 31.5% required dialysis and 12.4% died. These figures point to the significance of this neglected public health issue.

Snakebite treatment according to the biomedical management protocol includes AV, which is essential and the only antidote for envenoming, and supportive treatment such as airway management, treatment of hypotension and shock, treatment of acute kidney injury, management of hemostatic shock and treatment of the bitten site with antibiotics if needed [20]. In Myanmar, government doctors and paramedical staff are trained to provide treatment with AV and supportive treatment.

In Myanmar, treatment of snakebite is impaired by problems with the supply of AV, and by shortage of adequately trained staff, particularly in rural areas. These limitations may contribute to the persisting use of traditional methods. Use of the healthcare system in Myanmar is dependent on several factors, including cost, previous experience, fear of surgery, and belief in religious or spiritual healers. Additionally, in some cases AV causes adverse reactions [7, 21]. Hence, the communities may also harbour fears of biomedical treatment.

Many traditional healing methods, such as local incision, herb ingestion, application of snake stones, and tattooing, are ineffective, and in some cases, harmful [7, 10]. Their use can cause infection, bleeding, gangrene and other problems. In this way, the use of traditional healing may further delay or complicate necessary biomedical treatment.

With the continued use of traditional healing practices, it is important to develop a better understanding of the nature of healing practices, the communities’ reasons for and views about its use, and the interface between traditional and biomedical components of the health system. In many snakebite-affected countries, an envenomed victim may need to walk (or be carried) for many miles to reach a primary health post. Gutiérrez and colleagues assert that ‘studies of the circumstances that delay the access of people bitten by a snake to health centres are of great value …… [and that the studies] should include in-depth analyses of the cultural characteristics of the communities, the way snakes and snakebites are perceived, the cultural background of local healers…..’ [22].

As part of a larger community and health services development project in Myanmar, the aim of this participatory action research was to engage with rural communities to learn from their perspectives, their health knowledge, and reasons for healthcare-seeking practices.

## Methods

Using participatory methods, traditional healing for snakebite was studied through the lens of community knowledge, experiences and traditions. Gutiérrez et al. note that modern health programmes in rural communities are often culturally biased and paternalistic, lacking participation of the community in question [23]. Participatory methods acknowledge that local communities have valuable stores of knowledge which can guide development [24, 25]. Learning from communities through participatory approaches is even more important for issues which affect impoverished people. Snakebite is a problem that mainly affects impoverished rural people, and its neglect at the global level is largely due to the fact that the affected populations lack political voice [26].

Participatory rural appraisal sessions (PRA) were organised in three communities in villages in Kyaukse and Madaya townships of Mandalay Division. They included the creation of ‘problem walls’ to reflect what the community saw as problems they faced. Focus Group Discussions with three groups of health care providers were conducted in the same settings. The primary care workers, which included Health Assistants, public health staff and midwives, are responsible for basic curative care at community health centres. They provide vaccinations, outreach, public health, preventative and health promotional activities in community and home settings.

The three communities and the health care providers in those areas were selected considering representation of various areas of the township, distance to services in the city, access to care, and logistics and feasibility. Three participatory appraisal sessions took place, in 2016, in public local community meeting places, ensuring an appropriate environment that fostered maximum comfort and interaction between the participants. 135 participants took part in PRA sessions that were held between 10 am and 2 pm.

The majority of participants attended for the whole session, whilst others joined late or left early due to obligations such as work and family. This flexibility is part of the participatory and empowerment process, and contributions were respected and considered even if community members were unable to participate for the whole duration of the session. The communities were approached through primary health care workers, who, after permission from village leaders, invited individuals to participate. No community members or primary health care workers refused to take part and no one withdrew from the research. Table 1 informs about the selection process.

**Table 1:** Selection process, number of sessions and participants.

	Participatory Appraisals	Focus Group Discussions
Sessions	3	3
Participants Number	135	23
Selection Criteria & Process	Representation various areas of the township, distance to city hospital, access to care, and logistics and feasibility Project staff approached the local PHC workers → PHC workers sought permission from village leaders → Communities approached through local PHC workers and village leaders → invitation to the whole community + invitation to snakebite victims → invited community members requested to ask other community members	All primary care staff at the health centre where PRA was conducted
Participants	Community women and men in three villages and adjoining villages	Primary Healthcare staff at the government health centres
Work	Men mostly farmers Women farmers & home makers A traditional healer	Midwives, Health Assistants, Public Health outreach staff
Snakebite victims among the participants	7	0
Victims who sought care from a traditional healer	5	-

After introducing the learning aims of the sessions, community members were encouraged to decide among themselves the most important aspects of the snakebite problem and issues with health care for snakebite in their own and surrounding communities. Throughout this process, participants were also encouraged to share their personal and family experiences. Community members discussed, defined and wrote key issues and problems on the flip charts in the shape of bricks (problems). They then discussed the solutions that would be needed to ‘break the wall down’.

In the same villages where the PRA took place, FGDs were conducted at the government rural health centres with 23 primary care workers, with a focus on the extent of snakebite problem in the area, use of traditional and biomedicine health care by the locals and the reasons for such use. These health staff had experiences of snakebite either personally or through their work.

In addition to the communities noting their issues on flip charts, a scribe took notes of the discussions. Each of the participatory appraisals and FGDs yielded several pages of raw narrative data. The data were then analysed to identify themes. The thematic analysis consisted of 6 phases [27], the first step of which was familiarisation with the data through reviews of the detailed notes taken at PRA and FGDs as well as the visual data such as problem walls. The next stages in this analysis involved generating initial codes and interpretative analysis of these codes, leading to searching for themes i.e. important patterns and concepts relevant to the research question about healing methods and the reasons for their use. Themes were then reviewed and refined by carefully considering relevance to the main research questions, whether identified themes were backed by sufficient data, and whether there was clear distinction between the identified themes. The themes were then defined and reported for discussion. This analysis focused on patterns of use, type of traditional methods and the reasons rather than on prevalence. Therefore, such thematic analysis did not include counts or statistical analysis.

The research was conducted with ethics approval from the Human Research Ethics Committee at the University of Adelaide and Ethics Committee at the Department of Medical Research at the Ministry of Health in Myanmar.

## Results

### Context

Kyaukse and Madaya townships are farming communities. All community members who participated in the discussions and appraisals acknowledged that snakebite was a problem in their communities. Working on farms and or walking to or from farms were the activities associated with snakebite, particularly early in the morning and in the evening. People informed that the harvest times in this region, June-July and October-November, were associated with higher incidence of snakebite. The community members considered snakebite a major health issue, and emphasised the need for further inputs for prevention and improved curative care. They informed about the inadequate health awareness and difficulties in accessing transport. They had good knowledge about preventive methods, particularly the need to wear boots when working in the fields and to have a torch while working at night. However, they informed that many do not practice these preventive methods for cost and convenience reasons. For example, it was mentioned that the boots are costly, hot to work in, and get stuck in the mud causing the work to slow down. The solutions by the community members included the need for further health education, access to less costly or subsidised appropriate boots, adequate supplies of AV at health centres closer to their villages, and better access to transport. In fact, groups of local volunteers across many communities facilitate transport for patients from their villages. For example, one of the communities where a PRA session was held had a community car that the locals use to transfer patients to hospitals. However, that car was in need of repair when PRA took place.

Healthcare providers considered that with increased use of mechanised farming, the snakebite problem was decreasing. Most bites were by Russell’s vipers, and a few by cobras and a variety of other species. Healthcare providers informed us that access to health services had improved in the last few years as a result of better transport.

### Use of a combination of traditional and formal biomedical care

Firsthand accounts by those who had been bitten by snakes informed us that the use of traditional healing in these communities was either as a stand-alone treatment without using biomedical care, or in conjunction with biomedical care. Those who used traditional healing in conjunction with biomedicine did so before or after the biomedical care. Seven of the PRA participants shared some information about their experience and use of health services and traditional healing. This information is summarised in Table 2.

**Table 2:** Information provided by the snakebite victims.

Age when bitten	Gender	Bite Site	Location	Snake identity	Hospital use	Traditional healing used	Additional comments
35	M	Foot/lower leg	Turmeric plantation	Not known	Yes	After hospital	Was not fully healed after hospital. Said that he became fully fit only after seeing a traditional healer
45	M	Foot	Banana plantation	Viper	Yes	No	Had a torch but was not looking down, was not wearing shoes.
18	F	Finger	Betel plantation	Viper	No	Yes	Not known
28	M	-	In the village	Not known	Yes	After hospital	Bitten whilst getting water to fields
20	M	Foot	In the village	Not known	Yes	Yes	Not known
12	F	Leg	In a house	Not known	No	No	Snake was under her bed
Multiple times	F	Various	Plantations	Not known	Yes	After hospital	Treated at hospital. Said that she became fully well after receiving treatment from a traditional healer

Two victims informed us that they had visited a traditional healer after presenting to a hospital. Another two said they had been to a hospital but did not use the services of any traditional healer, and the other three said that they had been to a traditional healer but not to a hospital or rural health centre. One woman reported being bitten on her finger while she was picking betel leaves when she was 18 or 19 years old. She went straight to the monk for traditional treatments in the form of herbal medicine and tattooing. She said that she was fully healed after a month of that treatment. One man working in his turmeric plantation at dusk was bitten by a snake and fainted. He was driven to Kyaukse hospital where he was admitted, received AV and discharged after two months. Details of his hospital treatment were not discussed as the focus of discussions was traditional healing. After discharge from the hospital he went to see the monk for further treatment.

### Traditional healing methods

With regards to types of traditional healing, the community members did not distinguish between traditional healing as a practice and traditional healers as the practitioners. Traditional healing was seen both as a profession and a tradition. However, in reference to the methods being used, community members made a clear distinction between monks and other traditional healers as two separate types of traditional healer. This distinction appeared to be due to the spiritual healing aspect of the care provided by the monks.

A range of methods of healing were used by both monks and other traditional healers. Both practiced physical and spiritual methods. The physical techniques included asking the patient to chew the root of a particular plant to diagnose what type of snake had bitten the patient. The diagnosis was based on taste; whether it was bitter or not. Other practices included making incisions with a razor blade, tattooing with either ink or herbal medicines, use of a syringe to suck out venom, and rolling a heated glass bottle on the bite site to draw snake venom out. Tying a rope or a piece of cloth above the bite site as a tourniquet is practiced by many snakebite victims and the community as a first aid method, noted through observation of patients at the Project’s site hospitals. Communities did not discuss the tourniquet at the PRA sessions as a traditional method; probably because of the fact that health care providers at health centres and hospitals now advise communities not to use a tourniquet (a recommended first aid method for snakebite about a decade ago).

**Fig 1:**
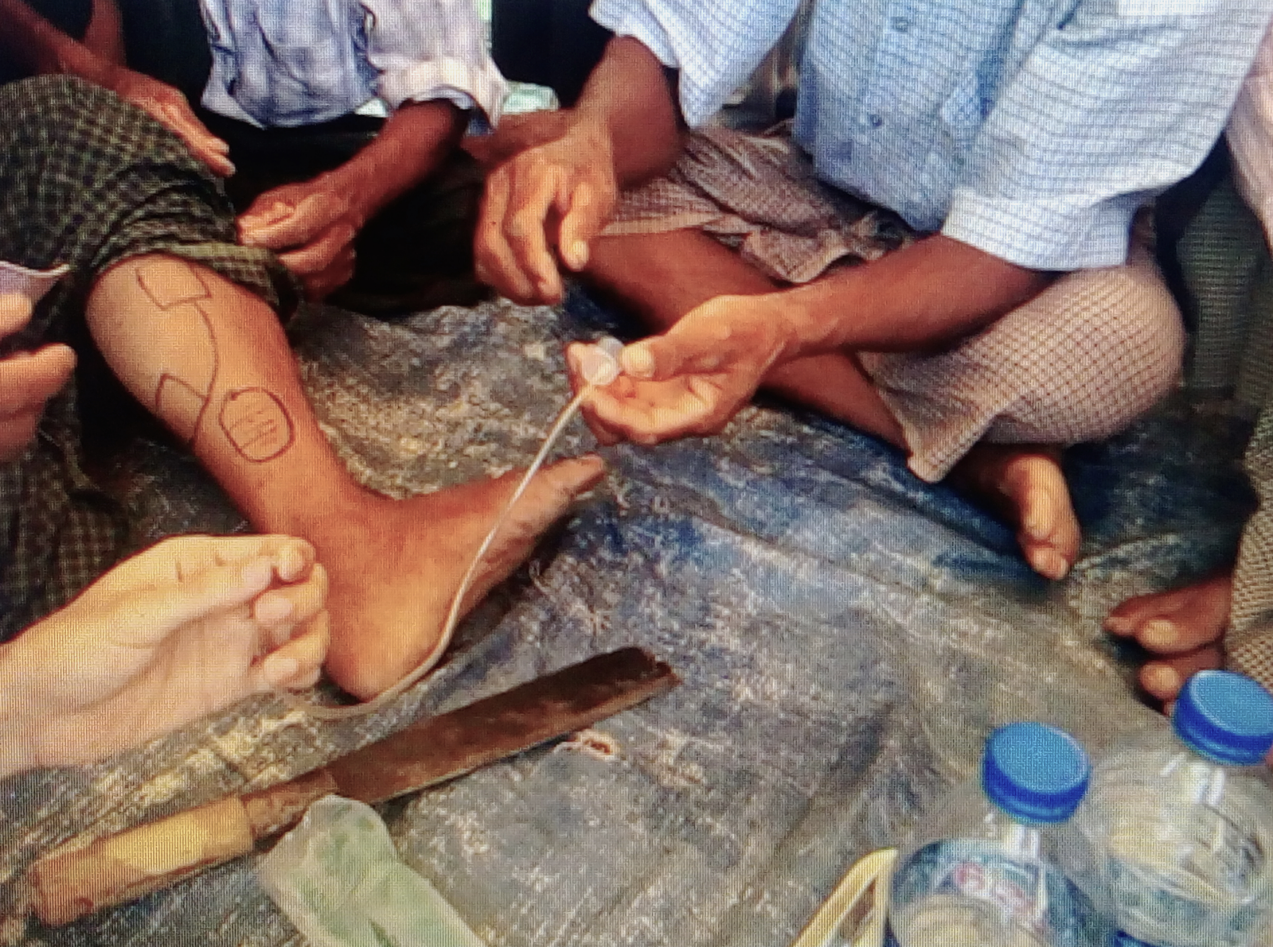
Community members illustrating traditional healing methods. Some of the participating community members are shown in the figure illustrating a particular method where traditional healers cut the area bitten by a snake with a blade and try to extract poison using a syringe.

Faith-based spiritual techniques used by the monks and by the other healers included use of holy water, chants, prayers and astrology. Community members mentioned that “herbal concoctions” are used by both monks and other traditional healers. The following is a description, as reported by a community member, of one of the methods used by a monk:

“Using a razor blade, the monk makes 10 parallel surface cuts around the wound. He then takes a 20cc plastic syringe and cuts off the top, placing it on the bite site. Using a second syringe and a thin tube he draws out the poison, which can be seen being removed in thick clots (Fig 1). After the poison is removed, blood starts to come out of the syringe. If the blood is not clotting, the monk knows [that he needs] to refer patients to hospital. The monk uses a bowl of bottled water to flush out the syringe throughout the procedure, and has the patient consume some traditional medicine [recipe unknown to the community members] to increase urine output. If the patient urinates 3 times [after the treatment], they are said to be cured”.

How the traditions are passed on, and the methods used, was elaborated on by a 58 year old local traditional healer who lives in the village and participated in the participatory discussions and analysis:

“When I was 33, I myself was bitten by a snake and went straight to a monk for treatment. The monk tattooed my entire body with medicine and gave me coconut juice infused with herbal medicines. I recovered after 3 days. After this, I was taught by the monk and then I decided to learn more [about traditional healing for snakebite]. The monk also used astrology. For example, determining the type of treatment needed by a patient by considering the geographical location where a patient was born. He has now stopped practicing, and refers some snakebite patients to me. I have been practicing since 1997 and have treated more than 50 people for snakebite, all of these have been successful. I use astrology and some traditional remedies”.

The staff who participated in FGDs were also aware of these treatment types, including tattooing, incision, and using a glass bottle to remove venom.

### Reasons for the use of traditional healing

The quality of traditional healing was perceived to be good and was trusted by the participants. The community members reported that visiting a monk or other traditional healer was common and most of the snakebite victims in these communities access such services at some point following the bite. Many participants were of the view that even if the local health centres were adequately stocked with AV and if the treatment was available close to the village(s), people would still choose to see a traditional healer in addition to using biomedical care. In contrast to the community members’ views, the healthcare providers said that if there was enough medicine available at the centres, most people would report straight there and not to a traditional practitioner, despite strong community belief in traditional healing for snakebite.

According to community members, one reason for trust in and use of traditional healers is that deaths from snakebite after treatment by traditional healers are rarely seen by the communities. They reported success rates of up to 95% among those who use the local traditional treatments. Communities also held the monks in high regard due to the spiritual and community service aspects of the healing, and lacked access to formal biomedical care because of high cost, distance from health care facilities, and difficulties with transport. For community members, the cost of care was a major factor. Most pointed out that the cost of hospital treatment for snakebite, including car hire, food for carers, accommodation for carers and some out of pocket medicine was around 300,000 Kyat (about 220USD). In contrast, treatment from a monk or traditional healer was often free, for a voluntary donation, or minimal fee. One person informed that they paid approximately 30,000 Kyat (22USD) for their visit to the monk. One village had no car and for any emergency the villagers needed to rent a car at a high cost. In the two villages that did have community cars, one had broken down and the community lacked the funds to repair it.

Another reason community members gave for using traditional healing first was the desire to avoid visiting hospitals, with some speaking unfavourably of the treatment in hospital settings. They cited factors such as unkind treatment and being afraid of the staff. In contrast, others said that they liked the care provided at the hospital. In fact, one of the community members bitten was very satisfied with the treatment he received at hospital, and as a result, did not seek any traditional treatment.

The misconception that snakebite victims cannot be treated at a hospital or health centre without bringing the snake involved with them was voiced by two of the community members who had been bitten, one of whom had tried to catch the snake after being bitten. It is true that accurate identification of the offending snake can be useful, particularly if the snake is a non-venomous or minimally venomous species, thus allowing the snake-bitten person to be reassured and discharged from treatment in most cases. At some FGD sessions, staff noted that if the snake was clearly a non-venomous or minimally venomous species, they could then discharge the patient without need for referral on to a larger health facility. These staff appeared confident in their ability to identify such non-risk snakes.

The healthcare staff mentioned transportation, low cost of traditional healing and strong traditional beliefs as reasons for the continued use of traditional healers. Some staff emphasised that traditional beliefs, not cost, were the major reason. The healthcare staff themselves did not appear to believe in traditional healing, and stressed that they had no contact with the traditional healers or monks about the treatment of snakebite patients. The healthcare staff also considered inadequate staffing and AV supplies at the health centres as contributing to the use of traditional healing. At one of the sessions, the staff said that the local monk only treated people because he wanted what was best for the community and that he knew that the local rural health centres were not adequately stocked with AV. Adding to this, some staff suggested that monks and other traditional healers could possibly be incorporated into the modern healthcare system.

## Discussion

Traditional beliefs, proximity to care, low cost, and perceived or actual inadequacies of the formal biomedical healthcare system emerged as the main factors associated with the local communities’ use of traditional healers. Spirituality also plays a key role in influencing decisions about treatment, and services by monks are seen as associating care with spirituality and community service. These beliefs appear to have a major influence on the use of traditional service despite awareness among community members that treatment is available in the formal biomedical sector, that the formal care is heavily subsidised and that many patients indeed have used formal biomedical care. The trust in and use of traditional care for snakebite is continuing despite expansion and better access to and use of biomedical care [28]. In fact, around the globe, the overall use of traditional and alternate medicine has increased in the last decade within the context of escalating costs of care and an increasing emphasis on patient-centred care [29].

Traditional healing plays a positive role in terms of social connectedness, harmony, services and support for fellow community members. However, there exists a knowledge gap which needs to be addressed to facilitate better health and wellbeing. Results of this participatory assessment indicated that one of the reasons communities trust the capacity of traditional healers is a perceived better success rate of the treatment in the traditional sector. In fact, a significant number of snake bites are either dry bites by venomous snake species with no systemic envenoming, or bites by one of the many species of non-venomous or minimally venomous snakes. Many who visit traditional healers as their first point of care therefore may view their traditional treatment as having been successful after suffering only from a dry bite or non-venomous bite. Additionally, as traditional healers often refer patients to hospital only when the symptoms are severe, hospitals end up seeing more patients with clinical complications, many of whom deteriorate and die [30]. These factors combine to generate a misperception about success of traditional healing and ineffectiveness of services in the hospitals. This suggests a need for health education for communities to bring about informed choice. Another misconception that community health education programs should address is that of the need to bring the dead snake to the hospital for identification, in order for diagnosis and treatment of the patient. Efforts by the patient or community members to kill and bring the snake could lead to further harm, either by the snake, or through further delays in necessary AV treatment. However, as noted earlier, identification of the snake can be beneficial in some circumstances. The increasing availability of mobile phones with inbuilt cameras, even in rural communities, might provide a future avenue for snake identification without a need to capture/kill the snake.

Some felt that the traditional health practitioners simply want what is best for their patients, and would happily forego their practice if formal services were more available. In fact, they informed that healers and monks do already refer severe snakebite cases to formal biomedical services. At the same time, we received the impression that some monks and traditional healers relied on snakebite treatment as a form of income, which would mean that foregoing treatment of snakebite victims could affect their livelihood. One strategy could be to integrate traditional medicine into the national health systems and define how it might support disease prevention, promotion and treatment [29]. This research informed us about the communities’ trust in the service provided by the traditional healers, and for that reason we believe that the traditional healers could be engaged to provide community health education and appropriate first aid and facilitate early transfer of patients to nearby healthcare facilities. As some of the traditional healers provide care on a fee for service basis, identifying mechanisms to financially compensate traditional healers could facilitate their engagement for community health education, appropriate first aid and timely referrals. The role of traditional practitioners in working with skilled care providers and facilitating referrals to hospital is well investigated for other highly important public health issues such as safe motherhood services. For economic, access or cultural reasons, many women receive care from traditional birth attendants. As deliveries are safer if conducted by a skilled attendant or at a facility providing quality care, integration of traditional birth attendants with the formal system has been promoted. One of the barriers to this integration is the potential financial implication for the traditional care providers [31]. Research, however, has highlighted that the traditional care providers are willing to work with the formal health services [32]. As the use of services provided by traditional healers is common among snakebite victims, it is important that efforts are made to engage the traditional healers; and it is anticipated that they could be willing. However, as provision of care to snakebite victims is a source of income for traditional healers, a strategy that does not address financial concerns may fail to create an effective linkage between snakebite traditional healers and the formal health sector.

While it could be argued that some traditional healing practices should, at the very least, be discouraged as being harmful and delaying referral to medical care, this participatory analysis of the local situation suggested that ‘phasing out’ of the whole concept of traditional healing for snakebite would not be an easy or advisable option. Policies that intend to force such moves may be challenging due to deeply held beliefs and a myriad of factors influencing peoples’ attitudes and practices, but more importantly, may also create tension and conflict between the formal health sector, communities, and traditional healers. Conversely, the concomitant use of biomedical care and traditional care by these communities offers an opportunity to facilitate the involvement of traditional healers for improved preventative practices, correct first aid procedures, and timely access to AV and other biomedical care as needed. In Nepal, for example, it was found that the traditional healers could be successfully trained to perform critical roles in primary prevention, first aid, and referrals [13].

Though disregarding traditional healthcare altogether may seem logical from a clinical point of view, it fails to take into account complex societal and health system factors. Even worse, such an approach tends to undermine cultural and traditional beliefs, and could cause further alienation and disempowerment. Within a context of deeply embedded beliefs, more credible are those suggestions which promote a dialogue between traditional healers and modern medical practitioners [8, 2, 33]. A plan to combat the snakebite problem needs to acknowledge traditional healers as an important stakeholder with potential to act as partners in prevention, appropriate first aid and prompt referral to effective treatment at health facilities. With monks providing traditional healing to the communities in Myanmar, they could be engaged in a similar way to the Maw Phra, or Doctor Monk, program which was implemented by Dr Prawase Wasi during the 1970s and early 80s. The program involved Buddhist monks, who are highly respected in Myanmar, and hold a strong association between Sadha, education and care for patients. It provided refresher courses in herbal medicine, as well as some basic skills of biomedicine [34].

Limitations: This research was able to yield some valuable data, particularly about the methods of and reasons for the use of services by traditional healers. Nevertheless, it has some important limitations. First, while local staff were actively involved in facilitating these sessions, interpreters were required for the researchers who didn’t understand Myanmar language. The local staff interpreted and translated; however, some interpretations may have been lost in translation. Secondly, this research was limited to three communities only, and the perspectives that we have gained about cost, transport, effectiveness of biomedical care could be area specific. Thirdly, the participatory sessions took place during the middle of the day when some young farmers had to be working in the fields. Since they are a demographic at such high risk of snakebite their participation would have been valuable. Fourthly, some of the healthcare staff had interacted separately with the project team members as a part of the wider project, and their views, particularly about the need for AV to treat snakebite patients, might have been influenced by that interaction. Despite these limitations, the purpose of this research was to highlight the snakebite phenomenon from the communities’ point of view. Public health and health system managers should take into account the valuable perspective that was gained, and would stand to benefit from discussions with their local communities on how the biomedical and traditional systems might operate side by side.

## Supporting information

S1 Checklist STROBE checklist. Checklist of items for this observational study.
(PDF)

S1 Data FGD and participatory sessions notes. This file contains sessions’ notes including information on number of participants, community and service settings, and concepts discussed by the community and health care providers at the participatory sessions and focus groups discussion sessions.
(PDF)
