# Supplementary material for: Why snakebite patients in Myanmar seek traditional healers despite availability of biomedical care at hospitals? Community perspectives on reasons
Source: PLoS Negl Trop Dis. 2018 Feb 28;12(2):e0006299. doi: 10.1371/journal.pntd.0006299 (PMC5847227; doi:10.1371/journal.pntd.0006299)
Supplement: S1 Checklist — Checklist of items for this observational study. (PDF) [file pntd.0006299.s001.pdf]

Traditional Healing for Snakebite in Myanmar

STROBE Statement —***cross-sectional studies***

	Item No	Recommendation
**Title and abstract**	1	(*a*) Abstract – lines 31-50
		(*b*) Abstract – lines 31-50
**Introduction**		
Background/rationale	2	Introduction - Lines 66-142
Objectives	3	Introduction - Lines 144-156
**Methods**		
Study design	4	Method – lines 159-174
Setting	5	Method - lines 176-181 & Results lines 227-231
Participants	6	Methods – lines 183-193, 202-205
Variables	7	Methods – Lines 195-200
Data sources/ measurement	8*	Methods – lines 207-219
Bias	9	Limitations – lines 461-476
Study size	10	Method – 178-181
Quantitative variables	11	Not Applicable
Statistical methods	12	(*a*) Describe all statistical methods, including those used to control for confounding
		(*b*) Describe any methods used to examine subgroups and interactions
Not applicable		(*c*) Explain how missing data were addressed
		(*d*) If applicable, describe analytical methods taking account of sampling strategy
		(*e*) Describe any sensitivity analyses
**Results**		
Participants	13*	Methods – lines 13-189, Methods lines 202-205
		(b) Give reasons for non-participation at each stage – Not applicable
		(c) Consider use of a flow diagram – Not applicable
Descriptive data	14*	Methods – lines 192-200, Methods lines 202-205, Results – lines 227-249
		Methods – lines 192-200, Methods lines 202-205
Outcome data	15*	Not Applicable
Main results	16	(*a*) Results – lines 227-374
		(*b*) Report category boundaries – Not Applicable
		(*c*) Estimates of relative risk - Not Applicable
Other analyses	17	Not Applicable
**Discussion**		
Key results	18	Discussion – lines 377-408
Limitations	19	Limitations – lines 461-476
Interpretation	20	Discussion – lines 412-445
Generalisability	21	Discussion – lines 412-445, Limitation – lines 461-476
**Other information**		
Funding	22	Funding by DFAT, Australia. Information provided online.

*Give information separately for exposed and unexposed groups.

Note: An Explanation and Elaboration article discusses each checklist item and gives methodological background and published examples of transparent reporting. The STROBE checklist is best used in conjunction with this article (freely available on the Web sites of PLoS Medicine at http://www.plosmedicine.org/, Annals of Internal Medicine at http://www.annals.org/, and Epidemiology at http://www.epidem.com/). Information on the STROBE Initiative is available at www.strobe-statement.org.
