# Supplementary material for: Why snakebite patients in Myanmar seek traditional healers despite availability of biomedical care at hospitals? Community perspectives on reasons
Source: PLoS Negl Trop Dis. 2018 Feb 28;12(2):e0006299. doi: 10.1371/journal.pntd.0006299 (PMC5847227; doi:10.1371/journal.pntd.0006299)
Supplement: S1 Data — This file contains sessions’ notes including information on number of participants, community and service settings, and concepts discussed by the community and health care providers at the participatory sessions and focus groups discussion sessions. (PDF) [file pntd.0006299.s002.pdf]

Focus Group Discussion

KTD RHC

Facilitators:

Dr. Afzal Mahmood, Mya Myint Zu Kyaw, Eliza Schioldann, Tissan Tnd (Intern)

Nyien Nyien Aung (Translator)

Participants

Health Assistant – KTD RHC

MMM: PHS II, KTD RHC (working here since 3 years)

ZLN: PHS II, KTD RHC (2 years)

CS: PHS II, K – Sub-Centre (3 years)

HLN: PHS II, LMC Sub-centre (3 years)

AMS: PHS II, YS Sub-centre

FGD Notes

Introduction and greeting

First, MMK explained the need and process for the consent and the nature of FGDs as being a 2 way discussion [rather than question answers]

We will focus on the snakebite problem. What is your opinion of snakebite in this area?

- Approximately 5-10 snakebites in 46 villages this year
- Most go to a traditional healer

(Health staff were then asked about average snakebite incidence, in their respective areas)

- DCS: "My area consists of 8 villages, there is about 5-10 snakebites per year"
- One of the PHS II originally from Madaya- still has family there who report increasing snakebite in the last year. She has referred 3 cases to hospital
- DHLN: Looks after 5 villages, there are 1-2 snakebite cases per year
- DZLN: Looks after 14 villages, 2 cases in the last month
- Total population of these villages combined = 37117, approximately 7648 households

As healthcare staff could you please share some snakebite [related] experiences with us?

- In this RHC, ASV is limited, so we've been informed that after administering 2 vials, we must refer to hospital." (This has recently changed to 4 vials). [One dose for Russell Viper envenoming is 8 vials – more than one dose may be required]
- Today, the RHC has 8 vials: 4 from India and 4 from MPF."

- Snakebites are mostly from vipers, not so many cobras
- Many bites from unknown snakes
- At the RHC, the HA has administered ASV for the last 3 years

Can you share with us your experiences of snakebite prevention and management, and if these have been successful?

- We need more health education and more health staff
- [people] Need money for rubber boots
- Many farmers own their own rubber boots but do not wear them because of the hot weather
- Most of the health staff agreed that people did not wear boots because of the heat. [However the HA told an incident where a man managed to prevent a snake fangs from penetrating because he was wearing rubber boots]
- Community experience has revealed to us that even with preventative measures such as rubber boots, people still get bitten (e.g. on the hand).
- People go to the monk because you can donate [in return for receiving the care] what you [patient/family] like
- Many people are very interested in community education and are also requesting ASV

Staff agreed on 3 main issues:

1. Bites [occurring] despite prevention [practices are used increasingly]
2. Visits to the monk [for healing]
3. Inadequately stocked health centres

So why do the communities not use these preventative measures such as rubber boots?

- People like to finish their work on time, boots slow them down, especially if they have to work in water and mud.
- At night, people do use torches
- Some snakebite victims don't care about the risk of snakebite as they do not want to modify their lifestyle; carrying a torch is inconvenient.
- [After some discussion] all of the health care staff agreed that many people do use torches at night. The use of torches is increasing (up to 90% of farmers), but the use of boots seems to be decreasing.

Traditional healing

- DCS: "n my area there is a monk, but if the case [envenoming] is very serious, the monk refers [the patient] to hospital

- Reasons [for community members to use traditional healers' services]:
    - Closer
    - Cheaper
    - Monk cures most cases
- In our village there is a traditional healer and people come to him from other villages
- The monk and the traditional healer use some of the same methods, e.g. the glass bottle being used to remove the venom
- Sometimes they test where the venom is with his finger and then decides where to cut
- Monk uses the tattooing method
- This year, the HA has given antivenom to 3 people; 1 of them had been to the traditional healer first

A facilitator checked with the healthcare staff that they said that the main reasons for seeing the traditional healer were cost, distance, and beliefs.

- The community knows that there is not enough ASV in the village so they don't come to the RHC, and they can't afford to go to the hospital
- They [community] believe they will be cured only after going to a traditional healer"
- Another agreed, stating that "the high success rate of the traditional healer sets a good example"
- People go [visit traditional healers] because they can save time and money
- There is an overall community belief that the traditional healer successfully heals people

What was the snakebite situation 5 years ago?

- Some of the staff said that they have only been in their positions for 3 years so they do not know
- [The facilitator requested the staff to share experience from personal perspectives as they have lived in these communities for much longer]
- Situation is getting better because now we have a community car. Before, cars were only privately owned and not for emergency use
- Number of snakebites has decreased
- Tractors on the farm (and other modern technologies) have reduced the number of encounters with snakes
- In the past, we lost so many lives to snakebite because ASV was unaffordable and we had to go to the traditional healers
- Previous [in some cases] health assistants used to keep the ASV for their own personal use

- In the community, it is easy to share knowledge, so successful hospital cases can change community perceptions
- However, if people see the traditional healer successfully curing people, it is also likely they will still see the [traditional] healer

You said lives are being saved because people don't have to go to the traditional healer, so why are people still going?

Scenario:

- 5 people go to hospital
- 4 get cured, tell their friends
- 1 has a bad experience or is not cured- also tells their friends.

So we see that this dissemination of information can work in both a positive and negative way

What constitutes good treatment?

- Kind staff & adequate supplies

How can we encourage hospital attendance?

- Sufficient Antivenom
- Sufficient staff

What [will be the perception of the community and staff] if we have enough ASV and the [some] patient still dies?"

- "We would accept this"

But earlier you mentioned that the bad results spread bad news?

- Yes, but if we know that staff treated the case as well as they could and the patient still died, he is just unfortunate. The problem is when ASV is given but the staff manage the case badly.
- Other staff agreed. ASV is one thing, but the quality of care is also important

We now know that many still go to a traditional healer. We've asked what to do to make less people go to a traditional healer.

Can you see a positive future role for the healer?

- If there were enough ASV, monk would refer to the rural health centre
- Staff inform that the reason the monk treats people is because he knows the RHC is not adequately stocked with the necessary ASV
- Staff are unsure if the traditional healer feels the same way
- Monk cures because he sympathises with the community. If there were a better way, he would be happy to change his role

Is there a role for traditional healing in the healthcare system?

- Let the traditional healer know that we have ASV (inform)

- We can inform the healer, but do know not how he will act
- Educate the community that there is enough ASV
- We think that people will stop going to traditional healers/monks if they have education and ASV.
- We also have a problem with lack of staff. Staff get transferred and then are not replaced, so we are overloaded with patients."

The facilitator assured that the points shared by the staff are very important and that the snakebite project can provide support [training etc] and [help] talking to the people who have the decision making capacity [for improved supply of antivenom, training, community work etc]

What should be organised now:

- Pamphlets for education
- Fridge (maybe? – for better cold chain)

Personal observations:

- In general, the FGD went well and most people seemed to be participating. Some of the participants were shy at first. MMK encouraged that all should feel welcome to contribute. The group were always respectful to each other and listened carefully to what others had to say before stating their own opinions.
- On some matters such as torches, there was some initial disagreement and people talking over each other, but this was resolved and the healthcare staff were able to come to a general consensus on the issues being discussed.
- The food being given to the healthcare staff during the session rather than after appeared to be a good approach, as healthcare staff were starting to lose concentration, and after eating seemed to be re focused
- As opposed to the FGDs in the other 2 villages, there were only healthcare staff present, and no community members in close proximity to listen to the discussion. This may have allowed the healthcare staff to speak more freely with us, as they did not have to be careful about what they were saying.

Focus Group Discussion

Date 16 July 2016

TY RHC Staff, FG discussion in PL Village

Facilitators:

Dale Halliday, Mya Myit Zu Kyaw, Eliza Schioldann, Nyien Nyien Aung (translator)

Participants:

5 Midwife (3-33 years experience, up to 18 in current posting)

1 Public Health Staff Level 1 (3 years experience)

1 Health Assistant (34 years experience, 4 years at the current post)

Introduction and Greetings

<u>What do you think about snakebite in the community?</u>

- In some areas [in the catchment of this health centre] snakebite is still a problem but in other areas it is quite rare
- Based on the [recent] survey there hasn't been much snakebite
- 10 snakebites in the last 30 years
- From 2015 to now [July 2016], 33 animal bites. 5 snakebites, 28 scorpion and others
- The staff conducted a community survey and responses are not indicating that snakebite is a big problem

<u>What do you think about snakebite as a problem?</u>
<u>Are there less bites than before?</u>

- One staff mentioned: 3 bites in her time, refers victims to Kyaukse hospital. 1 patient: 14 year of age girl: died after late presentation to hospital. 1 was bitten on the finger last year
- She can't do much to help them but shares her knowledge of identifying where the bite is from a snake, scorpion etc.

  - Patient drove himself on moto to hospital

- “Most people in this area go directly to hospital”

- “Community know what to do when they are bitten by snakes”
  - Another staff:
    - Did not refer patients to hospital.
    - Her 2nd patient went to a traditional healer (“fake doctor”).
    - Used Antivenom 8 vial dose
    - After a week, patient died
    - Patient did not go to the HA

HA:

- Another Staff: My daughter had a snakebite, went to Mandalay General Hospital. Died after 3 days
  - Treated 10 cases in his last posting
  - Checks symptoms & clotting time, performs first aid

One of the area [has more snakebites]:

- 3-4 snakebites per year
- Min Ye hospital 12 miles away

<u>What about traditional management? Which does the community prefer?</u>

[Community members] Prefer hospital, but some go to the monk because of:

- Saving money
- Saving time
- Snakebite victim’s own beliefs

- Cost:
  - If they don’t have enough money they have to see the monk
  - People think ASV is too expensive
  - Last August (2015), there was a snakebite case that went straight to hospital and cost 1 mill. kyat (1135 Aus $)
  - This word spreads & deters other people from hospital
  - They don’t think about the seriousness of the bite, they are only thinking of the money

- Referrals have been made but people didn't go because of transport/money

- Stories of success are shared [by the snakebite patients] with others who then visit the traditional healer

[Do people consider that] if they spend [more] money, they could be alive [by having treatment, if needed, with antivenom]?

- "Now that we know this, we do refer straight to hospital"
- "In my area, people go to the monk.. it's close to the road so easy to access

- Influencing factors:
  - Financial
  - Transport
  - Traditional beliefs

- Based on these 3 [factors as listed above], people decide where to go
- "These days, transport is better so people prefer to go to hospital"
- "20 years ago, we used homeopathic methods but not anymore"

What can we do to educate the community?

"We have to keep in touch with the community, give more talks"

"Educate"

"Help them understand"

- In 2017, they hope to have a clinic in the area [this village]
- "The person who helps the community must have good qualities"
- Education must happen at the schools and health centres
- "This information will be spread"
- "Combine talks about snakebite with other issues such as dengue fever"

Can we find out ways to lessen preference for traditional healers?

Availability of ASV

More health education

Within the next 10 years, how do we stop people from relying on the traditional healer? Can we work with him [traditional healer]?

- He does have the knowledge to refer people to hospital if the case is serious enough
- To get the traditional healer to refer people we must befriend him first”
- To share our abilities with his abilities
- Help the community to recognise dangerous practices
- Also to educate the traditional healer: his methods can be dangerous to himself as well
- Improve relationships between healthcare staff and the community, then they are less likely to go to a traditional healer”

- Healthcare staff should be:
  - Well qualified
  - Good at communication
  - Available at all times

- Once I was late to see a patient so it created misunderstanding between me and the community”

- Foster mutual understanding between health staff and community”
- Especially when we are busy and the community have urgent needs

So if there was a clinic here, people wouldn’t go to the traditional healer?

If the clinic was here that’s where people would go

If we have these things, will people still go to the traditional healer?

- Not if people know ASV is available
- They will change their traditional beliefs
- The community has a social welfare group which meets once a month. At these meetings they could spread information on snakebite
- Community has a community vehicle that can be used for transport of snakebite patients
- For all ailments, people used to use traditional healing because of lack of money and transport. But now, they go to hospital for everything

The process of referring patients to hospital has become easier. It was difficult in the past
It takes time

Is the ASV available at the sub-center [close to the village]?

No, just at the RHC

At meetings, we could share information about ASV availability"

I could spread information at 4 villages, but how do I use these tools?... I'm 100% sure that in my 4 villages, people won't go to a traditional healer if modern medicines are available."

Focus Group Discussion

6/7/2016

RCH at village SM (village name abbreviated for confidentiality)

Facilitators: Dale Halliday, Eliza Schioldann, Nyien Nyien Aung (translator)

Participants: 10 healthcare workers

Experience ranged from 2-27 years

FGD took place after a health education session [for the community]

Introduction and greetings

Is snakebite a big problem in this area?

- No, it's quite rare - about 1-2 snakebites a year (in the catchment area of this rural health centre)
- 3 snakebite cases this year, 2 of them died
- "1 died because they used traditional methods before going to hospital"
- "Our survey work has revealed that snakebite is not really a problem"

Information about traditional Healing in the area

- More people use traditional methods.
- The healthcare staff work in 34 villages:
- 14 [villages] use traditional healers/monk
- 20 [villages] use modern medicine
- There is 1 monk and 3 non religious healers between the 14 villages
- "The use of traditional healing for snakebite is increasing"

Why do people use a traditional healer?

- Easy transportation
- Cost of care
- Strong beliefs

Traditional healers:

- 2 of the healers are the monk's students
- They [offer holy] drink water or use the 'cutting' procedure
- No knowledge dissemination [by traditional healers] for preventative measures

- No contact between traditional healers and healthcare providers
- The healthcare staff only know what the villagers tell them about traditional healing
- In the last 2 years: 5 snakebites
- 2 [treated at] hospital (both died)
- 3 [used] traditional healer (2 died, 1 lived)
- One of the victims went to a traditional healer before they went to hospital
- [health care staff believe] Villagers have a strong belief in the traditional healer
- [Healthcare staff say] they do not believe in the traditional healers/monks
- Monk refers the more serious cases to hospital

Could the monk be trained to work with modern medicine, since he is a trusted member of society?

The monk is old, but his students might possibly be interested

What is the solution? What can we do as healthcare providers to get people to go to hospital?

- [the group discussed the unavailability or inadequate availability of antivenom and services and suggested] If there is enough medicine at the hospital, we think most people will go to the hospital and not the traditional healer

Physical issues such as transport can be solved, but what about spiritual/belief issues? How can we educate people/change their perceptions?

- If the medicine is available in [the village], maybe they will go to the subcenter
- It will take time
- Snakebite survivors who share their stories after positive experiences in hospital could be a good influence
- For snakebite, traditional beliefs are still strong so people go to a healer, but for other things they go to hospital
- The older [more experienced] healthcare staff informed that they have seen a change [that more people using modern medicine]
- Snakebite is rare
- Health education and information sharing will eventually be successful

Observations:

- The healthcare staff were in the middle of carrying out surveys for the project so already had knowledge of snakebite incidence in the area. This may have influenced the direction of the FGD
- Staff were quite adamant that snakebite is not really a public health problem in the area
- The FGD took place in an open area with other community members listening, so staff may not have felt that they could speak freely about their opinions

Participatory Rural Appraisal

6/7/16

YB village;

SM RHC, Kyaukse

Session 1

Present

LHV, health care staff and village leader

Participatory Rural Appraisal 6/7/16

Ye Byie village; Sa Mar RHC, Kyaukse

Present: Dale Halliday; Eliza Schioldann, Sherrol Kaing (intern/note taker); Dr. Then Htun Oo (translator); Aunty K (Sa Mar LHV/co-facilitator); 50 villagers (40% male/60% female)

Introduction and greeting, PRA purpose and method

A village elder briefly introduced the village for information of field team

- Elder explained that the village is predominantly farmers, who grow onions, chillies and other vegetables. Is a larger village (~800pax), but started very small
- 1 snakebite death in last 15 years

The participants who had suffered snakebites then shared their experience

Man 1:

- 42 years of age
- Bitten many years ago, was emotional so somebody else told his story
- Bitten while getting water

Man 2:

- Bitten on the foot 10 years ago while walking in the village (there was no lights)
- Killed the snake
- He was miles away from the clinic and village but did not want his friends to help him so he walked himself, eventually hopping because of the pain in his foot
- Went to hospital
- Was still sick when he came home from the hospital so then he went to the monk

- Poison was removed by monk, but wound/infection (probably from cow dung) remained a problem

Woman 1:

- Bitten when she was 12 years
- 4am, snake was under the bed
- She passed out
- Her brothers and sisters took her to the clinic/hospital on a motorbike
- Stayed for 1 or 2 weeks, family also stayed with her for that time
- She had swollen leg for 1 month after being discharged from hospital
- Kept taking medicine that was prescribed and after 1 more month was fully recovered

P R O B L E M W A L L

community members discussed among themselves and considered snakebite and snakebite treatment related issues that negatively affect their health and healthcare outcomes – and constructed a wall with each problem representing a brick

The community split up into 3 groups- one with men, one with women, and one mixed group. All 3 groups generally came up with the same problems, mainly focusing on transport.

- **Transport:**
    - The village does not have cars, so if someone gets bitten they must rent a car from another village
    - The monk lives in the village so transport for traditional healing is not an issue
    - If they are on motorbike, and the family goes with the snakebite victim to hospital, there are often 3-4 people on one motorbike

- **Lack Of Community Education**:
    - They do not know the first aid necessary when someone has been bitten, other than (splint?)
    - Also have not been taught anything about prevention of snakebite

- **Lack Of Electricity:**
    - Cannot store ASVs, no lighting... (see man 2)
    - Sa Mar has no electricity

- **<u>Low Efficacy Of ASV:</u>**
    - The ASV they have used has not been effective.. bad quality

- **<u>Cost:</u>** Estimated total cost (including car hire, hospital admission, ASV)= 300000 kyat (334 AUD)

One person from each group then presented group's ideas. Session had been going for about 2 hours and people started to leave, but everyone that stayed was engaged and interested.

The participants then mentioned treatment by a local Monk

- Monk: 70 Years of age
- Also treats for rabies
- 3 days a year he trains younger monks who are allowed to practice in other areas/villages
- They [local community members] trust the monk more than biomedicine: 1500 people have been treated by the monk for snakebite and only 5 of them have died
- They say that he successfully cures (90-95%) of snakebite cases, referring the others to seek health treatment.

Traditional method [the participants informed about the methods used]**:**

1. [The Monk traditional healer in this area] Using a razor blade, makes 10 parallel surface cuts around the wound.
2. Takes a 20cc plastic syringe and cuts off the top
3. Connects a 2nd 20cc syringe to the cut-off top using a thin tube
4. Places cut end of syringe on the bite site
5. Sucks out the poison with the 2nd syringe:
    a. Can see poison coming out in thick brown clots
    b. Usually 5 clots are removed
6. Uses a bowl of bottled water to flush the syringe/funnel a number of times throughout the procedure
7. Gets patient to drink 1-2 drops of traditional medicine (made by monk - recipe unknown) for urinal output

    ⇒ If patient urinates 3 x after, then they are cured
8. Can determine the type of venom by having them chew on a certain root.
    a. Bitter: Viper

b. Spicy: Cobra
c. Lemon: Scorpion (monk doesn't treat)
d. No taste: Recovered

- When blood starts to come out in the syringe: if blood is not clotting, refers to hospital (about 5% of cases)
- Procedure costs 30,000 kyat (33 AUD)
- Due to his reputation of so few fatalities, villagers place more trust in monk than in hospital
- When asked if they would still visit the monk 1st if a clinic was in the village, most replied yes, because he is reliable, and often people will visit him after the hospital if not fully recovered
- Used same procedure on patients who come from hospital

PRA Session 2

YB Village

Present: Dr. Afzal Mahmood, Dale Halliday, Mya Myit Zu Kyaw, Eliza Schioldann, Nyien Nyien Aung (Translator)

Men 32

Women 60

- Took place in the community meeting area
- Head of the village & public health staff present:
  - LHV not present due to injury
- Lunch break at school so there were a few children
- AM, DH, MK, ES & NA ate lunch provided by community & invited health staff to join
- After lunch, people trickled in
- At most there was:
  - ~32 men, ~60 women
- People began to leave after ~ 1.5 hours

- Introduction by the facilitator DH: recapped from the last PRA at this village, sharing that the last session the community helped define the problems, and that this second session is to take the next step i.e. defining solutions.

- Project staff had consolidated the problem wall/statements from the previous session into a new problem wall that was shown to the community

Issues summarised into 4 main categories:

1. Financial problems
2. Transport
3. Lack of education & knowledge
4. No well stocked clinic

  - Community agreed that this problem wall accurately reflected what they had discussed in the last session

- DH asked community to prioritise problems in order to identify solutions/take action
- Community responded that all problems were of importance
- Facilitator agreed, but explained the reasons for prioritisation:
    - Some challenges/problems easier to address
    - Some can be addressed at the community level
    - Others need to be addressed by health, government, other departments
    - "If the problems are easy, let's fix those first. If everyone agrees to focus on the same problem first, let's do that"

Facilitator asked about the decision making process by the locals in that community. The participants informed that the decision are made by discussing things together.

- Community discussed problems as a group, deciding their **biggest problem** is transport difficulties (i.e. vehicle & quality of roads) causing delays in access to care
- [and discussed the] Most manageable problem= lack of a well stocked (with anti venom), accessible clinic in the village

The participants were then requested to define solutions in terms of what can be done about the snakebite problem and access to care by the community,
What can be done by the government, and what the snakebite project could do.

The participants were split into 3 groups based on where they were sitting & provided with paper/markers for the activity:

- Group 1: "Brothers"- men only
- Group 2: "Sisters"- women only
- Group 3: "Brothers & Sisters"- mixture of men & women

- 25 minutes of discussion within the groups, (with occasional prompts by the facilitators): brothers & sisters group was very active, brothers group equally active.
- However, sisters group was not as active [in discussion]
- Somewhere in the process, a 4th group organically formed [some participants from the three groups came together to form this fourth group]

- At completion, a member from each group stood at the front and presented their group's ideas to the community.

Group 1:

1. **Place for the clinic**
    - The community would help to find a location
2. **Staff**
    - All of the staff are government, could we train some young local people from the village?
    - We have a mobile clinic but it only comes once a month
3. **ASV**
    - Need support as it is quite expensive
4. **Transport**
    - We have some but not enough
    - Even if there is a clinic here we still need transport for emergency cases

Group 2

"Everybody knows that the problems are a building for the clinic, staff, and medicines"

1. **Clinic**
2. **Staff**
3. **Medicines**
    - These all require money, so we need support
4. **Transport**
    - For better roads, we have asked for help from the government
    - This will happen in budget year 2016-17
    - At the moment, we are contributing personally

Group 3

1. **Clinic**
    - "We can survive if we have a clinic, but we need help to get the land."
2. **Transportation**
    - Transport will help us save time
    - The vehicle has broken down
    - We need money, where can we get it?

Group 4

1. **Transportation**
   - We need more money- this could come from community support and from the government
2. **Clinic**
   - One participant's mother died from snakebite when she was 5, because the family didn't have money to go to hospital
   - If there had been a clinic in the village this could have been avoided

	Group 1	Group 2	Group 3	Group 4
**Clinic**	Place for the clinic: we would help to find a location	Need $$ and support	We can survive if we have a clinic, but need help to acquire the land	If there were a clinic in the village, snakebite deaths could be avoided
**Staff/First Aid Training**	All of the staff = government. Can we train some young local people from the village? Mobile clinic only comes monthly.			
**Medicine**	Need support to pay for ASV as it is quite expensive		Need enough ASV at the subcenter to save valuable time	
**Transport**	We have some but not enough. Even if there is a clinic here we still need transport for emergency cases	Better roads: we've asked the govt. for budget year 2017-18. At the moment, contributing ourselves	We have a mobile clinic but the vehicle has broken down.<br>Need $$ to fix the car	Better transport/roads. Need support from the community & from the government.

In listening to the 4 groups present, there was clear consensus about the main problems and their solutions. The village did actually have a community car that could be used to transport snakebite victims but that this car had broken down. The village has a car mechanic but is lacking the funds to repair the car. Already, a solutions was becoming clear for the issue of transport. All 4 groups pointed to the need for a clinic in the village, 2 of the groups stating that they would need help to find land. One suggested that this could be sourced from within the village by using resources, connections etc. They noted that even if there is a clinic in the village, transport is still needed for emergency cases. One group discussed issues relating to staff and to medicine can be merged into one problem.
Thus, the solutions relate to 3 areas:

1. **Clinic**
2. **Training**
3. **Transport**

1. **Clinic**
   - Ask the government [to open a clinic]
   - Look for a donor
   - Space for the clinic from the community?

The next step?

- Ask for help from the township development office

Facilitator AM asked who among the community could interact with the township development office

- Head of the village and 5 others will go and ask for it

Facilitator suggested that maybe someone who has experienced loss from snakebite is a good person to be involved in discussing the local issues and potential solutaions with the development office]. & some active people in the community can write the application. Someone well connected [with the government department] knows who to ask for the land? someone good at public speaking can represent the community.

2. **Training**

It was discussed by the community, snakebite project staff how to proceed with the training. Snakebite project staff MK and DH can assist. It was decided to select [by the community] 5 women, 5 men that can be trained in first aid so that we have resources in the local community

3. Transport

- There is a car mechanic in the village
- It was discussed that the community car may cost around $300 to be repaired, community has already raised $60. The project staff suggested co-payment of 4200 by the project and $100 by the community, to which the participants agreed.

Next steps:

1. Budget plan (village head take responsibility)
2. Interaction between the Mandalay Region Department of Health and the community members from this village
3. Community make plan for around 10 people to receive first aid training from the Myanmar Red Cross (facilitated and funded by the Snakebite Project)
   - Could be trained in all first aid not just snakebite, e.g. asthma, heart problems

Additional notes:

- The 2nd PRA very successful & community was highly engaged.
- Significantly more people present than the first time despite the rain, which we thought might put some people off coming
- Suggests a positive reaction to the first PRA; snowball effect
- Community recognised that responsibility=mutual & they should not expect that the government/the project will just hand over the money that is needed to fix the problem
- Very responsive to the idea of co-payments: not wanting to get something for nothing
- Community successfully formed a 'Community Committee' of 5 people that will liaise with the project/represent the community

Participatory Rural Appraisal

12/7/16

MT village; TY RHC, Kyaukse

____

Mon Taing, Than Ywar RC:

30 Women

27 Men

Present: Dale Halliday, Eliza Schioldann, Mya Myint Zu Kyaw, Dr.

Then Htun Oo (translator), Kyauk Win (HA from Than Ywar RHC)

[3 community members shared their snakebite incidences]

Man 1:

- Bitten 5 years ago working in a turmeric plantation at 7PM
- Was walking around the plantation, bitten by a snake & then fainted
- Couldn't move so was driven to Kyaukse
- Was given some injections of ASV
- "I was in hospital for 2 months"
- "I wasn't fully recovered when I was discharged from hospital"
- Snakebite site not healed
- Man believed that he had renal dialysis treatment twice but Health Assistant said that this was not the case
- "When I was discharged from the hospital I received some traditional treatments"
- "I used traditional treatments for 1 month afterwards"
- Monk provided treatments far from the village
- Ointments that have antivenom effects
- Hospital stay 300,000-370,000 Kyat
- 1 vial ASV= 70,000 Kyat
- First 4 vials free, he had to pay for the subsequent 3
- The monk provides treatment for free but you can give donations if you want

Man 2 (from a different village):

- Bitten this year, 30th April at 6:30PM
- Working on a banana plantation
- Was at the banana plantation with his family but left due to bad weather and came back by himself

- "I had a torch but was not looking down"
- Wearing no shoes
- Stepped on a viper: saw it with the torch afterwards
- Snake coiled up but didn't run away
- "I used banana leaves to kill the snake and took the snake back to the small hut on my plantation"
- Went to Kyaukse hospital by car: 45 minutes
- "I passed out in the car and woke up in the hospital at 9pm"
- Was given injections but didn't know what of
- Terrible pain but wound was in good condition
- 3 days in hospital
- Not allowed water so could not urinate
- Loss of appetite
- Kyaukse Hospital referred him to Mandalay General Hospital (MGH) where he spent 10 days because he needed dialysis
- He liked the care given at MGH
- While he was in the renal dialysis unit his foot became very swollen and blistered
- Was given dialysis 6 days after the bite
- Was then referred to the orthopaedic unit where he asked if he could return to his village
- After returning to his village on May 12, he had to return to hospital for 1 month
- He now has follow ups every 10 days at MGH because the wound is not healing properly
- Received a skin graft
- Total cost: 2,500,000 Kyat (2800 AUD)
- Has not used any traditional methods/seen a traditional healer

Lady 1:

- When she was 18/19 years of age, she was picking betel leaves and bitten on the finger by a viper
- Was treated and cured by a monk, who tattooed her arm with medicines
- Treatment took 1 month

P R O B L E M W A L L [community members discussed among themselves and considered snakebite and snakebite treatment related issues that negatively affect their health and healthcare outcomes – and constructed a wall with each problem representing a brick. The participants were divided into three smaller groups]

Group 1 [listed the following problems]:

- Family burden: Hard to make money so they work early in the morning and late at night: dangerous for snakes
- Expensive ASV
- Hospital staff don't treat people nicely
- Visiting a traditional healer first; causes delays
- Lack of appropriate footwear
- Late arrival at hospital
- Snakes hidden in the bushes; bushes should be cleared
- Lack of knowledge
- Walking carelessly without looking down

Group 2: [listed the following problems]:

- No clinic in town
- When bitten, they can't walk very far
- Lack of first aid knowledge so they require first aid help from other villages
- Families don't know how/cant afford to care for patients
- Lack of footwear

Group 3:

- Side effects of ASV
- Need to take snake to hospital for diagnosis
- Low efficacy of ASV
- Only monospecific ASV

Traditional Healer [share his story and treatment method**]:**

- 58 years old, lives in the village
- Was bitten by a snake himself when he was 33
- Monk tattooed his entire body with medicines & gave him coconut juice infused with herbal medicines
- Totally recovered after 3 days
- Was taught by the monk and then decided to go to Bago to learn more

- Monk also uses astrology: geographic location may determine treatment e.g. if the patient was born in the West
- Monk has stopped practicing so refers people to the traditional healer
- Traditional healer teaches younger people his practices
- Has been practicing since 1997 and has treated more than 50 people for snakebite: all successful
- Uses astrology
- Different chants for different days (sometimes uses religious chants)
- If pain is high, he will refer to hospital
- Gives medicines (secret recipe) with edible oils
- Recognised by Association of Traditional Medicine
- Believes that increased mental power and meditation will help him to cure patients
- After chanting and giving herbal medicines he watches the patient for 5 minutes
- After 15 minutes, he asks where the pain is and if it has spread
- If the pain hasn't spread, he sends the patient home with some herbal medicines
- After they vomit, there is a 75% chance they have been cured
- If not cured: Gives medicines every half an hour until they vomit
- One girl received medicines every half hour for 5 days before she was healed
- 3 herbal remedies depending on the type of snake
- Also treats other minor ailments

Main problems [the three groups shared with all the problems they considered, and the whole larger group developed consensus after discussing these problems further**]**

⇒ Lack of prevention e.g. torches, rubber boots
⇒ Preventative cost of treatment
⇒ Distance to hospital
⇒ Lack of transportation
⇒ Low efficacy of ASV
⇒ Low availability of ASV
⇒ Need for diagnostic tools
⇒ Use of a traditional healer

PRA

16/7/2016

YT Village

Participants

[The group discussed the extent of the problem and highlighted the extent and issues]

- They have the most snakebite in this area
- Mostly a farming village: rice paddies
- Afraid of the hospital because the care is not good
- Financial problems [high cost of care which people cant afford]
- [perception that] "If people go to hospital for snakebite they normally die"

<u>[The participants informed about the local traditional health and his practices]</u>

- He [traditional healer] makes an incision in the scalp (no matter where they have been bitten) and sucks the poison out with a glass bottle
- Takes about 5 minutes
- People from other areas come to this village to see the traditional healer
- Travel up to 1 hour to see the healer
- First point of contact is always the traditional healer
- If serious: The traditional healer refers them to hospital (Mandalay General Hospital)
- If not serious: the traditional healer H uses traditional method
- Has [is able to] cures 99% of snakebite cases [who receive care by him]
- Traditional healer only treats snakebite
- Healer is 35 years old, learnt methods from his grandfather
- Depending on how the patient feels, they can sometimes go back to work straight away

A snakebite victim participant shared his experiences

"I have been bitten by snakes many times on the farm"

"I have been bitten more than 20 times, 1 of them was a cobra"

"If I go to the hospital I have to take the snake with me so I go to the traditional healer instead"

P R O B L E M   W A L L [community members discussed among themselves and considered snakebite and snakebite treatment related issues that negatively affect their health and healthcare outcomes – and constructed a wall with each problem representing a brick, and discussed reasons to the use of traditional health services.]

- **Financial:**
    - Have to spend a lot to go to hospital [hence, may use traditional healers]
    - Need to save up money
    - Traditional healer free of charge/ voluntary donation
    - If the case is quite serious we will still go to the hospital

- **Severity of the bite:**
    - The community member can't tell how serious the bite is: need the traditional healer [to assess the severity]
    - The traditional healer can heal most cases
- **Too many snakes:**
    - Many snakes, every time they breed they have around 40 snakes
- **Harvesting:**
    - Harvest season is the worst time for snakebite
    - 2 cases per day during harvest (June/July, October/November)

- Distance not a problem as they have a community car and motorbikes
- The only first aid they know is to tie up the bite with a rope

[facilitator] "If you could tell the severity of the bike would you still go to a traditional healer?"
Yes. The community members mentioned that the snakebite patients will still use the traditional healers

"What do you know about prevention?"

- "Since I have been bitten many times I sometimes wear rubber boots in the fields but they are very hot"
- Clear the areas

*This PRA session was combined with a child vaccinations session
